# Mathematical modelling of reversible transition between quiescence and proliferation

**DOI:** 10.1371/journal.pone.0198420

**Published:** 2018-06-01

**Authors:** Nishtha Pandey, P. K. Vinod

**Affiliations:** Center for Computational Natural Sciences and Bioinformatics, International Institute of Information Technology, Hyderabad, India; King’s College London, UNITED KINGDOM

## Abstract

Cells switch between quiescence and proliferation states for maintaining tissue homeostasis and regeneration. At the restriction point (R-point), cells become irreversibly committed to the completion of the cell cycle independent of mitogen. The mechanism involving hyper-phosphorylation of retinoblastoma (Rb) and activation of transcription factor E2F is linked to the R-point passage. However, stress stimuli trigger exit from the cell cycle back to the mitogen-sensitive quiescent state after Rb hyper-phosphorylation but only until APC/C^Cdh1^ inactivation. In this study, we developed a mathematical model to investigate the reversible transition between quiescence and proliferation in mammalian cells with respect to mitogen and stress signals. The model integrates the current mechanistic knowledge and accounts for the recent experimental observations with cells exiting quiescence and proliferating cells. We show that Cyclin E:Cdk2 couples Rb-E2F and APC/C^Cdh1^ bistable switches and temporally segregates the R-point and the G1/S transition. A redox-dependent mutual antagonism between APC/C^Cdh1^ and its inhibitor Emi1 makes the inactivation of APC/C^Cdh1^ bistable. We show that the levels of Cdk inhibitor (CKI) and mitogen control the reversible transition between quiescence and proliferation. Further, we propose that shifting of the mitogen-induced transcriptional program to G2-phase in proliferating cells might result in an intermediate Cdk2 activity at the mitotic exit and in the immediate inactivation of APC/C^Cdh1^. Our study builds a coherent framework and generates hypotheses that can be further explored by experiments.

## Introduction

Tissue homeostasis depends on the ability of mammalian cells to reversibly switch between quiescence (G0) and proliferation. Cells remain quiescent in the absence of mitogen, contact inhibition and stress conditions. Under favorable conditions, cells exit quiescence and enter the G1 phase of the cell cycle, and become committed to the completion of the cell cycle. The decision to exit or enter quiescence is dysregulated in cancer and degenerative diseases[1, 2]. Therefore, understanding the molecular mechanisms that control the reversible transition between quiescence and proliferation is crucial.

A restriction point (R- point) in early G1 was defined based on pulsing the mitogen to mitogen-starved cells (G0). It is the time point when cells become irreversibly committed to the completion of the cell cycle in the absence of mitogen[3, 4]. The molecular mechanism associated with the R-point includes the regulation of transcription factor E2F by a pocket protein retinoblastoma (Rb)[5–7]. In the canonical view, mitogen induced expression of Cyclin D activates Cdk4 and Cdk6 (Cdk4/6)-dependent phosphorylation of Rb to release E2F[8–10]. In turn, E2F promotes the expression of Cyclin E, which activates Cdk2-dependent hyper-phosphorylation of Rb and full release of E2F. The double negative feedback loop between Rb and E2F mediated via Cdk2 is proposed to make the quiescence to proliferation decision bistable that ensures irreversible commitment to proliferation[11]. However, this view is challenged by the observations that hypo-phosphorylated/mono-phosphorylated Rb forms act as an inhibitor of E2F and hyper-phosphorylation of Rb is required for the E2F activation[12–16]. Further, the requirement of Cdk2 for the R-point passage is questioned by the observations that major phosphorylation of Rb occurs after the R-point and Cyclin E accumulation is detectable only after the R-point[17, 18]. However, it is also shown using the chemical genetics approach that selective inhibition of Cdk2 blocks R-point passage[19].

On the other hand, in some cycling cells, the mitogen sensitive R-point is shown to operate at the end of the preceding cell cycle (G2 phase) to control the proliferation to quiescence decision. In the presence of mitogen, cycling cells are found to bifurcate into two populations as they exit mitosis[20]. The majority of cells (80%) immediately commit to the cell cycle since they have Cdk2 activity at an intermediate level while other cells with low Cdk2 activity experience transient quiescence. In the absence of mitogen, cells exit mitosis with low Cdk2 activity to enter quiescence state and require mitogen for the cell cycle re-entry. Whether the R-point operates in the G1 or G2 phase is dependent on the cell type. The majority of cycling Swiss 3T3 cells exit mitosis with low Cdk2 activity (subjected to G1 R-point) compared to MCF10A cells, which show intermediate Cdk2 activity (subjected to G2 R-point)[20]. The proliferation to quiescence decision is controlled by the Cdk inhibitor p21. Endogenous DNA damage during the S-phase is shown to induce the synthesis of p21 in cycling cells[21, 22].

Further, a recent finding by Cappell *et al* (2016) suggests that APC/C^Cdh1^ inactivation at the G1/S transition serves as a commitment point that prevents re-entry into quiescence. This is based on the evidence that cells re-enter the quiescence state in the presence of stress after Rb hyper-phosphorylation and before APC/C^Cdh1^ inactivation. The cycling cells with intermediate Cdk2 activity immediately inactivate APC/C^Cdh1^ and enter the S phase[23]. The molecular mechanism proposed for the commitment at the G1/S boundary depends on the irreversible inactivation of APC/C^Cdh1^ by its inhibitor Emi1[23, 24]. Cyclin E:Cdk2 is shown to initiate APC/C^Cdh1^ inactivation before Emi1 promotes the accelerated inactivation. Studies have also shown that the proliferation to quiescence decision is controlled by double negative feedback loops between Cdk2 and p21, which control the degradation of p21 via the activation of two ubiquitin ligases, CRL4^Cdt2^ and SCF^Skp2^[21, 25, 26]. The regulation of APC/C^Cdh1^ and p21 is linked via APC/C^Cdh1^ dependent degradation of Skp2[27, 28]. Further, Dong *et al* (2014) showed that the levels of E2F increase in Myc-dependent manner and crossing a E2F threshold determines the cell cycle commitment. In this case, G1 Cyclins/Cdks control the timing of E2F crossing the threshold[29]. However, the inhibition of Cdk2 blocks APC/C^Cdh1^ inactivation and the S-phase entry[23].

How these regulations depending on mitogen and stress are integrated is yet to emerge. The complexity of the regulation coupled with different experimental set-up/cell type makes it difficult to understand this problem by intuition alone. The mathematical modelling approach provides a scope to develop a coherent framework that will help to generate hypotheses for further exploration by experiments. Previous studies on mathematical modelling of R-point and G1/S transition have shown the bistable activation of E2F and inactivation of p21, respectively[11, 21, 25]. R-point models show how cells emerging out of quiescence become mitogen independent and G1/S transition models show how p21 controls heterogeneity in cycling and quiescence states. It is important to revisit these models in the light of newer findings[13, 23, 30–33].

In this study, we developed a mathematical model to investigate the reversible transition between quiescence and proliferation in mammalian cells. The model integrates the current mechanistic knowledge and accounts for the recent experimental observations with cells emerging out of quiescence and cycling cells. We propose model scenarios for the cell cycle entry and exit with respect to mitogen and stress. We show that Cyclin E:Cdk2 couples Rb-E2F and APC/C^Cdh1^ bistable switches and temporally segregates the R-point and the G1/S transition. A redox-dependent mutual antagonism between APC/C^Cdh1^ and its inhibitor Emi1 can make the inactivation of APC/C^Cdh1^ bistable.

## Model description

**Fig 1:**
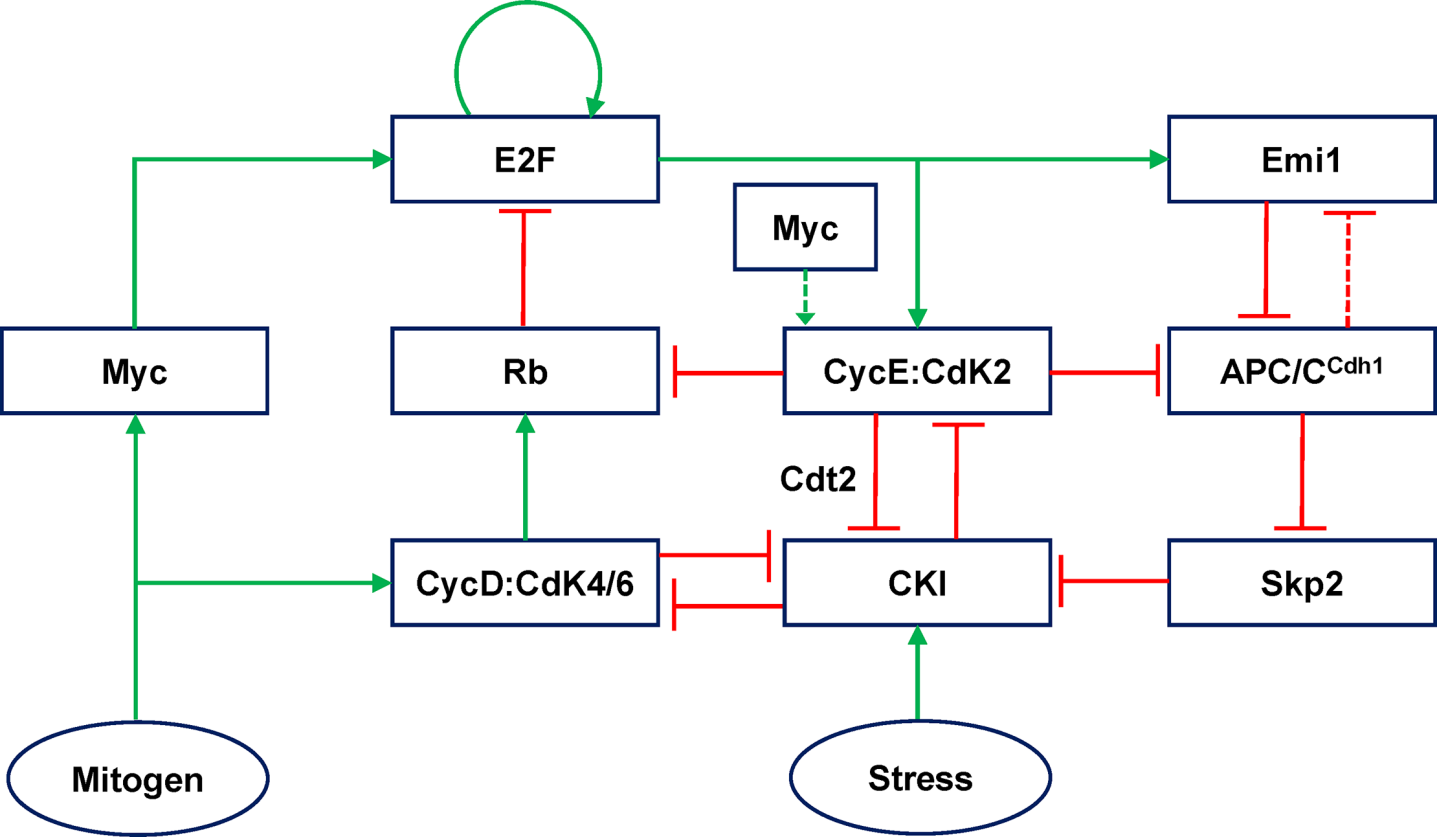
The molecular mechanism controlling the commitment points in the mid and late G1 phase of the mammalian cell cycle.

The model proposed here incorporates all the essential features controlling the reversible transition between quiescence and proliferation. At the heart of quiescence to proliferation transition is the regulation of Myc, E2F, Rb, Cyclin D:Cdk4/6, Cyclin E:Cdk2, APC/C^Cdh1^ and Cdk inhibitors such as p27 and p21 (represented as CKI) (**Fig 1**). Mitogen stimulates early and late phases of signalling events that promote Myc stabilization [34]. Though Myc is activated with early phase of mitogen signalling, the synthesis of Myc-dependent proteins occurs only with the late phase of mitogen signalling. This delay might be due to the presence of anti-proliferative signals, which have to be inhibited by the late phase of signalling. It is shown that p53-controlled anti-proliferative genes are induced by the early phase of mitogen signalling. This acts as restrain mechanism to prevent the commitment to the cell cycle until the late phase of mitogen signalling[35]. We consider mitogen as an input to the model which promotes Myc-dependent synthesis of Cyclin D, Cyclin E and E2F through the late phase of mitogen signalling.

In the model, Cyclin D:Cdk4/6 mono-phosphorylates Rb but this does not relieve the inhibitory effect of Rb on E2F. Rb inhibits E2F by forming a stoichiometric inhibitory complex (**S1 Fig**). Cyclin E:Cdk2 transforms the mono-phosphorylated form to hyper-phosphorylated form [13]. The hyper-phosphorylation of Rb is required for relieving the Rb inhibition of E2F. We also consider that Rb can be hyper-phosphorylated independent of mono-phosphorylation (by higher levels of Cyclin E:Cdk2 or Cyclin A:Cdk2). The release of E2F promotes its own synthesis and the synthesis of Cyclin E, Cyclin A and Emi1. This regulation raises interesting questions such as how E2F might be activated initially and what sets the time window for the hyper-phosphorylation of Rb. The hyper-phosphorylation of Rb/activation of E2F is controlled by CKI, which forms complex with Cyclin D:Cdk4/6, Cyclin E:Cdk2 and Cyclin A:Cdk2[36]. We consider that Cyclin E:Cdk2 is not only required for Rb hyper-phosphorylation but is also required for APC/C^Cdh1^ inactivation by phosphorylation. Further, Emi1 also inactivates APC/C^Cdh1^ by forming stoichiometric inhibitory complex (**S2 Fig**). Although Emi1 and Cyclin E are E2F targets, Emi1 accumulates only in the late G1 phase. Such a delay in Emi1 accumulation might be due to post-transcriptional/translational controls [37–39]. It has been proposed that Emi1 can be a APC/C^Cdh1^ target similar to Cyclin A[40]. Since Emi1 regulation in G1 is largely unknown, we hypothesize that Emi1 is an APC/C^Cdh1^ substrate and explore the dynamical consequences (**Fig 1**). SCF^Skp2^ and CRL4^Cdt2^ target CKI for proteasome dependent degradation in a Cdk2 dependent manner[41, 42]. The activation of these ubiquitin ligases occur post-APC/C^Cdh1^ inactivation since Skp2 is an APC/C^Cdh1^ substrate and CRL4^Cdt2^ dependent proteolysis is coupled to DNA replication via PCNA [27, 28, 43]. We consider that these ubiquitin ligases (represented as Ubl in the model) are directly regulated by APC/C^Cdh1^ similar to Skp2 regulation. We assume that the degradation of Cyclin E and E2F is promoted by the accumulation of Cyclin A [44–46].

Multi-site phosphorylation and dephosphorylation of Rb and Cdh1 are described using Michaelis-Menten kinetics and E2F dependent synthesis of components (Cyclin E, Cyclin A, Emi1) are described by Hill equation (n = 1), while all other reactions are represented by the law of mass action (**see S1 Supporting information**). The network was translated into a set of non-linear ordinary differential equations (ODEs), which describes the dynamics of individual components. Model parameter values were obtained by simulating the model to capture the single-cell dynamics of reversible transition between quiescence and proliferation in the presence/absence of mitogen and stress, and qualitative behavior of various perturbations [20, 23, 25, 29, 47] (**see S1 Supporting information**). We also performed one- and two-parameter bifurcation analyses to study the effect of different parameter values. The equations and parameter values are provided as part of the **S1** Supporting information along with the XPPAUT codes. Models were simulated numerically using XPPAUT, available from http://www.math.pitt.edu/~bard/xpp/xpp.html, to obtain the temporal profiles and bifurcation diagrams.

## Results

**Fig 2:**
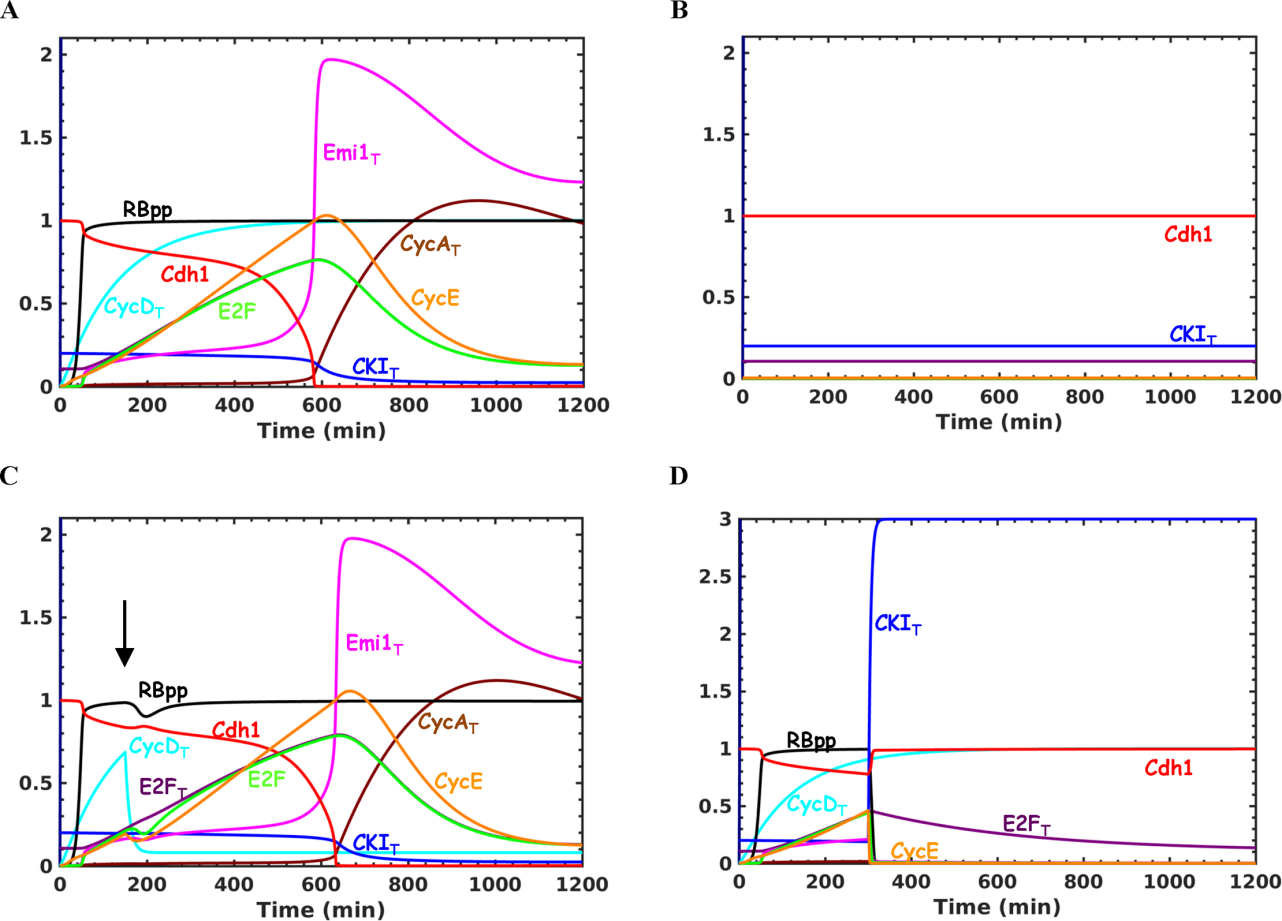
Temporal dynamics of quiescence to proliferation in the presence of mitogens. **(a)** Wild type, **(b)** in the absence of Cyclin D (k_scycdm_ = 0, k_scycds_ = 0), **(c)** inhibition of Cyclin D post-G1entry (k_dcycd_ = 0.1) and **(d)** in the presence of stress (k_scki_ = 0.6) before APC/C^Cdh1^ inactivation. Simulations are shown for S = 1 (mitogen level). The arrow represents the time of Cyclin D inhibition and exposure to stress.

Different model scenarios were considered based on initial signals (“trigger”) for E2F activation. Earlier models consider that Cyclin D:Cdk4/6 dependent phosphorylation of Rb relieves some E2F that promotes its further increase and Cyclin E accumulation. However, recent evidences show that Cyclin D:Cdk4/6 promotes the inhibition of E2F by Rb[13]. We explored how Cyclin D and CKI control the Rb-E2F switch under this circumstance. In the presence of mitogen, levels of Myc and Cyclin D increase (**Fig 2A**). Cyclin D overcomes the stoichiometric inhibition by CKI leading to mono-phosphorylation of Rb. However, the Rb hyper-phosphorylation can depend on the activation of existing Cyclin E:Cdk2 by the inactivation of CKI or/and on Myc- dependent synthesis of Cyclin E[48]. Similarly, E2F activation after Rb hyper-phosphorylation can depend on its initial levels at the time of Rb hyper-phosphorylation or/and on Myc-dependent synthesis of E2F[29]. In the first scenario (no Myc-dependent synthesis), Rb hyper-phosphorylation and E2F activation occur prior to the major accumulation of Cyclin E and E2F (**Fig 2A**), which is consistent with the experimental observations by Ekholm *et al* (2001). In the absence of Cyclin D, the exit from quiescence is blocked (**Fig 2B**). We also analyzed the Cyclin D requirement post-G1 entry. Hitomi and Stacey (1999) analyzed at what point in G1 the anti-Cyclin D antibody treatment becomes ineffective for cells emerging out of quiescence and cycling cells. We found that the inhibition of Cyclin D (mimicking anti-Cyclin D treatment) had no effect from the point when the Cyclin E exceeds CKI levels to sustain Rb hyper-phosphorylation and E2F activation (**Fig 2C)**. At very low CKI levels, this point coincides with R-point when cells become independent of mitogen. Therefore, we conclude that CKI levels determine whether mitogen withdrawal and Cyclin D inhibition become ineffective at the same or different time points in the G1. Such difference has been reported for cells emerging out of quiescence and cycling cells[47]. In the second scenario (E2F/Cyclin E is not present initially), the E2F (and Cyclin E) accumulation and Rb hyper-phosphorylation are delayed compared to the Cyclin D accumulation and Rb mono-phosphorylation (**S3A Fig**). The exit from quiescence becomes independent of Cyclin D (**S3B Fig**). The location of the mitogen sensitive R-point in these two model scenarios will differ if Rb hyper-phosphorylation and E2F activation is required for the passage of R-point.

**Fig 3:**
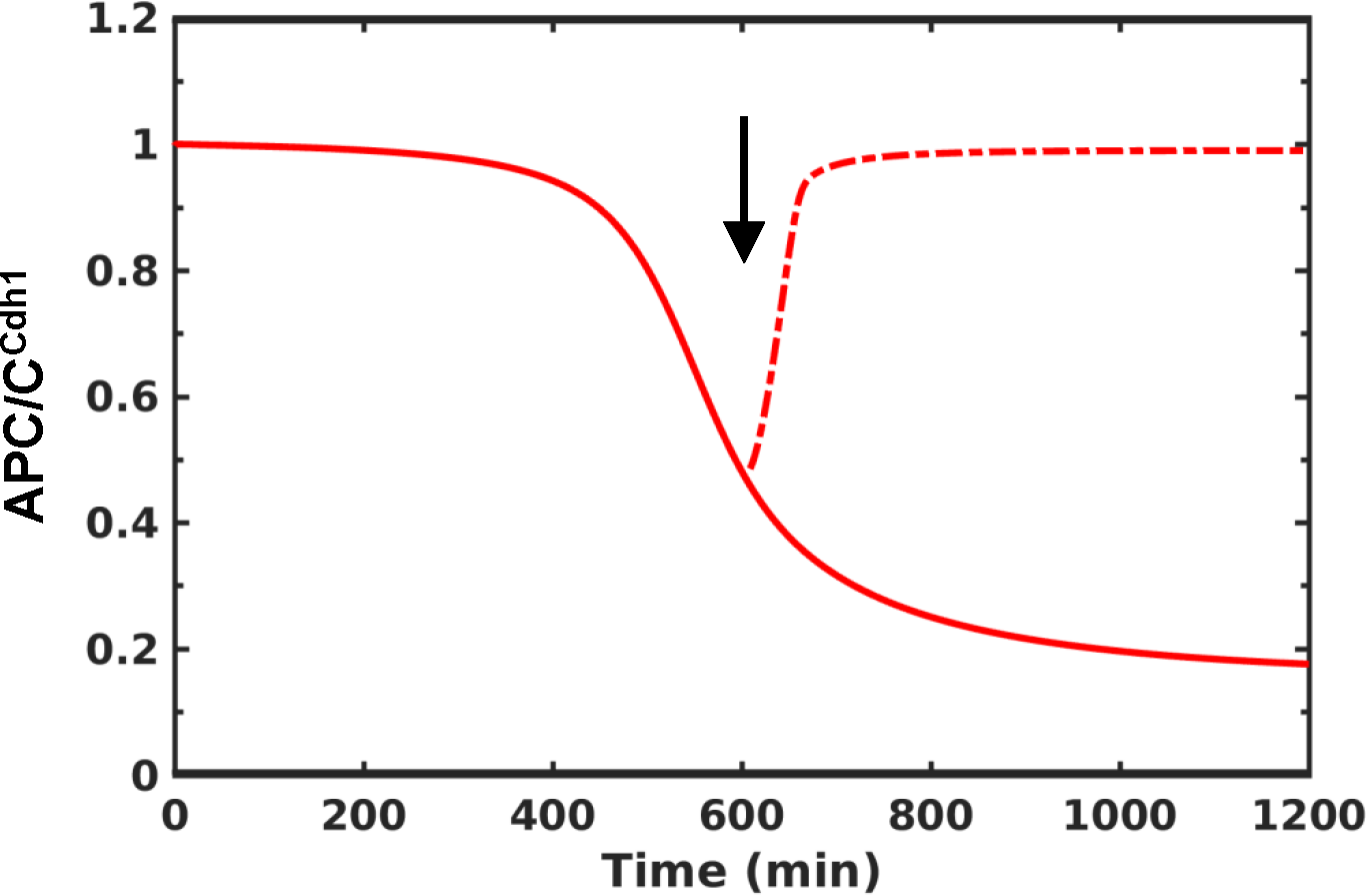
The dynamics of APC/C^Cdh1^ inactivation in the absence of Emi1. The dynamics is shown in the absence (solid line) and presence (dashed line) of Cdk2 inhibition (k_semi1_ = 0, k_dcyce_ = 0.01, k_dcyca_ = 0.01). Simulations are shown for S = 1 (mitogen level). The arrow represents the time of Cdk2 inhibition.

Further, we show that a rise in Cyclin E:Cdk2 activity (in E2F-dependent manner) initiates APC/C^Cdh1^ inactivation and accumulation of Emi1. The upregulation of Emi1 leads to accelerated inactivation of APC/C^Cdh1^ and degradation of CKI (**Fig 2A**). Here, the temporal separation between Rb hyper-phosphorylation and APC/C^Cdh1^ inactivation is achieved due to the low and high Cdk2 threshold requirement for the substrates, respectively. A slow rise in Cyclin E:Cdk2 activity is important for separating these two phases. A step-wise inactivation of APC/C^Cdh1^ is achieved by coupling Emi1 accumulation to the Cyclin E:Cdk2 activity dependent inhibition of APC/C^Cdh1^. In the presence of stress signals prior to the inactivation APC/C^Cdh1^, CKI levels increase leading to inactivation of Cyclin E:Cdk2 and Rb dephosphorylation (**Fig 2D**). On the other hand, APC/C^Cdh1^ inactivation leads to an increase in the degradation rate of CKI in the S-phase, which counteracts the CKI accumulation in the presence of stress making the G1/S transition irreversible (**S4 Fig**). In the absence of Emi1, APC/C^Cdh1^ inactivation slows down (**Fig 3, solid line**) and the inhibition of Cdk2 leads to the re-activation of APC/C^Cdh1^ (**Fig 3, dashed line**). This is consistent with the experimental observation by Cappell *et al* (2016).

**Fig 4:**
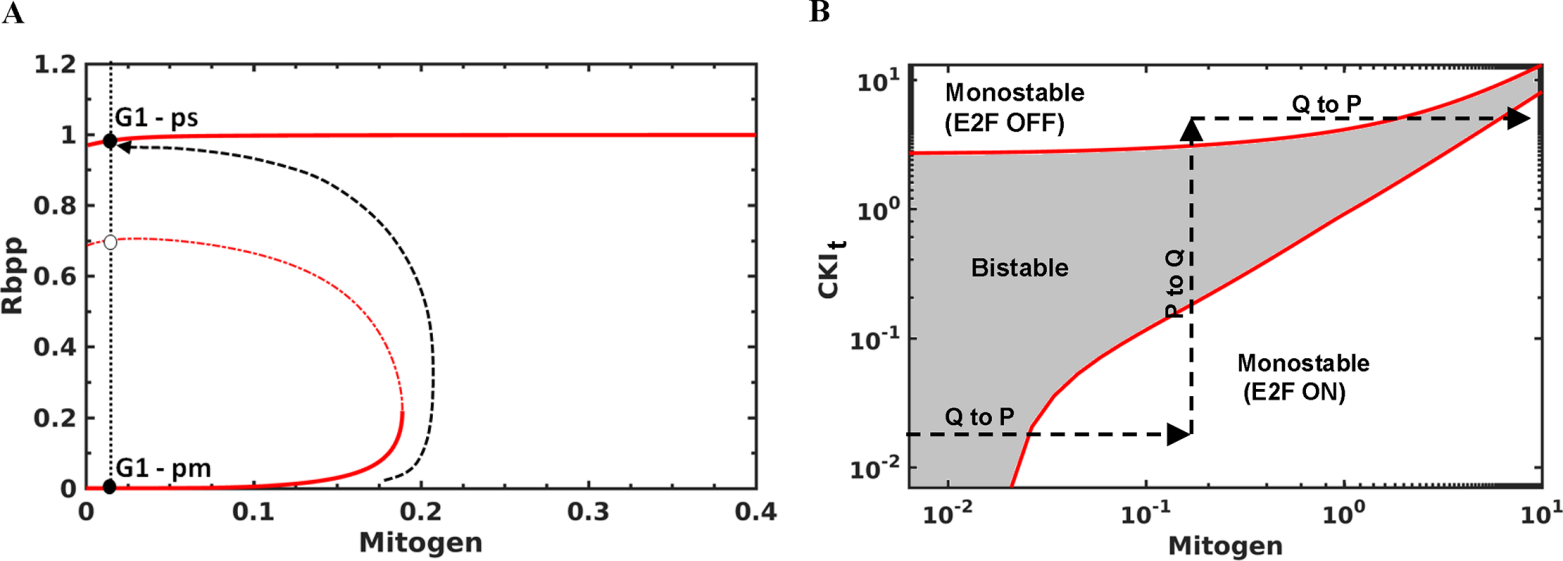
Bifurcation analysis of Rb-E2F subsystem. **(a)** The effect of increasing the levels of mitogen on Rb hyper-phosphorylation. Filled circle represents the stable steady state and empty circle represents the unstable steady state. The arrow shows the transition from mitogen- dependent to independent state. **(b)** Two parameter bifurcation diagram with different levels of mitogen and total CKI. The monstable and bistable regions are shown (E2F on and off). Q represents quiescence and P represents proliferation.

We performed the one-parameter bifurcation analysis of Rb-E2F regulation to understand the reversible transition between quiescence and proliferation. We show that the hyper-phosphorylation of Rb/activation of E2F is irreversible with respect to mitogen as observed previously [11](**Fig 4A**). At higher levels of mitogen, the system is monostable (high E2F state), while at low/intermediate levels of mitogen, the system is bistable (low and high E2F states co-exist). We studied the effect of increasing the CKI levels by performing the two-parameter bifurcation analysis. With increase in the CKI levels, as observed in the presence of stress, the system transits from a high E2F state via bistable region to a low E2F state (shown by an arrow in **Fig 4B**). However, this can be counteracted by increasing the levels of mitogen, which overcomes the CKI stoichiometric inhibition. In the absence of CKI, the system is bistable only for very low levels of mitogen and for intermediate and high levels of mitogen the system is in high E2F state. We also studied the sensitivity of Rb inactivation/E2F activation threshold to 5-fold change in parameter values by performing two-parameter bifurcation analyses. The Rb inactivation threshold is most sensitive (steep change in threshold for a small fold change in parameter values) to synthesis/degradation rate of cyclin E (k_scyceb_, k_dcyce_) and E2F (k_se2fb_, k_de2f_), and Rb total (**S5 Fig**). Further, the Rb inactivation threshold changes gradually (higher slope) with respect to CKI total and degradation rate of Cyclin D (k_dcycd_). On the other hand, the Rb activation/E2F inactivation threshold is sensitive (for less than 5-fold change) to the dephosphorylation rate of Rb (k_dprbp_) and shifts to the right (a positive value) bringing mitogen dependence with increase in k_dprbp_.

**Fig 5:**
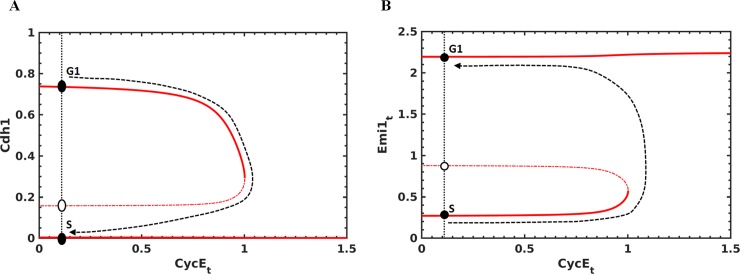
Bifurcation analysis of G1/S transition. The effect of increasing Cyclin E:Cdk2 activity on **(a)** Cdh1 inactivation and **(b)** Emi1 accumulation are shown. Filled circle represents the stable steady state and empty circle represents the unstable steady state. The arrow shows the transition from G1 to S-phase.

Further, we performed the bifurcation analysis of APC/C^Cdh1^ regulation at the G1/S transition. **Fig 5A** shows that the Cyclin E:Cdk2 activity initiates APC/C^Cdh1^ inactivation but is not required to maintain its inhibition. This depends on the Emi1 accumulation, which is irreversible with respect to Cyclin E:Cdk2 (**Fig 5B**). We also studied the sensitivity of APC/C^Cdh1^ inactivation threshold to 5-fold change in parameter values by performing two parameter bifurcation analyses. We observed that the APC/C^Cdh1^ inactivation threshold changes steeply with respect to the synthesis (k_smei1_) and degradation (k_demi1c_) rates of Emi1 (**S6 Fig**). The APC/C^Cdh1^ inactivation threshold also changes gradually with respect to activation (k_acdh1_) and inactivation (k_icdh1e_) rates of APC/C^Cdh1^. Further, the time window between Rb hyper-phosphorylation and APC/C^Cdh1^ inactivation is also sensitive to these parameter values along with the synthesis rate of Cyclin E (k_scyce_).

We also analyzed the behavior of cycling cells that sense the presence of mitogen during the G2 phase of the preceding cell cycle. It is shown that many cells exit mitosis with intermediate Cdk2 activity, keep Rb phosphorylated and increase the Cdk2 activity to commit immediately to the next cell cycle. On the other hand, a small fraction of cells enter a transient state of quiescence with low Cdk2 activity and Rb is dephosphorylated[20, 23]. We used the developed framework to understand the control of proliferation to quiescence decision by bifurcation in Cdk2 activity at the mitotic exit. We re-interpret the **Fig 2A** by shifting the Y-axis to an intermediate Cdk2 activity where Rb is hyper-phosphorylated to replicate the mitotic exit initial conditions (APC/C^Cdh1^ is active, Cdk2 is at an intermediate level and Emi1 is low) as observed in experiments. The position of Y-axis is chosen based on the experimental observation that cycling cells commit to the next cycle immediately (~ 4 hrs) by inactivating APC/C^Cdh1^ compared to cells coming out of quiescence (~ 8 hrs post R-point)[23]. The initial phase before the mitotic exit (left-hand side of Y-axis) is interpreted as G2/M phase with re-accumulation of Myc and Cyclin D in the presence of mitogen. Both of them are known to decrease after G1/S transition and increase in G2 only if the mitogen is present[32, 49, 50]. Similarly, in this framework, we assume that both E2F and Cyclin E also re-accumulate after they are degraded in the G1/S transition. In the absence of mitogen/Cyclin D, the re-accumulation of E2F/Cyclin E is affected and cells enter into quiescence after mitotic exit even in the absence of CKI. Further, increasing the CKI levels, mimicking the effect of increase in DNA damage that occurs naturally during the DNA replication, also promote entry into quiescence and this can be transient if the CKI levels decrease due to the DNA repair in G1. This picture suggests that an intermediate Cdk2 activity at the mitotic exit is due to an increase in Cyclin E levels through G2/M. However, the recent mapping of protein dynamics has shown that Cyclin E accumulates only after the mitotic exit, which contradicts our interpretations[32]. This can be reconciled if we hypothesize that there is accumulation of Cyclin E/E2F mRNA, which gets translated at/after the mitotic exit. E2F and Cyclin E levels decrease in S/G2/M due to Cyclin A:Cdk2 activation. However, a rapid inactivation of Cyclin A:Cdk2 at the mitotic exit might lead to a faster rise in Cyclin E that helps to maintain an intermediate Cdk2 state. This is also supported by the evidence that in cycling cells the accumulation of Cyclin E is driven by the re-accumulation of Myc in G2[48].

Another interesting observation that emerges is whether Cdk2 activity at the mitotic exit exceeds Rb hyper-phosphorylation threshold. This is assumed to explain the immediate inactivation of APC/C^Cdh1^ in cycling cells. Alternatively, increase in the rate of synthesis of proteins in cycling cells compared to cells emerging from quiescence can also explain the faster activation of Cdk2 and inactivation of APC/C^Cdh1^. The transcriptional difference can be attributed to the difference in the extent of chromosomal condensation in cells emerging out of quiescence and cycling cells[33].

## Discussion

A mathematical modelling framework was developed to analyze how Cyclin D and CKI control Rb-E2F regulation in two different contexts: quiescence to proliferation and proliferation to quiescence. We also explored how early (E2F activation) and late (APC/C^Cdh1^ inactivation) events of G1 are coupled to order the progression in cells emerging out of mitogen starvation and in cycling cells. We studied the dynamics in the presence and absence of mitogen and stress.

We showed that Cyclin D:Cdk4/6 by sequestering CKI tilts the Cyclin E:Cdk2 and CKI dynamic balance towards the activation of Cyclin E:Cdk2 and hyper-phosphorylation of Rb (**Fig 1** and **Fig 2A**).This occurs in spite of Cyclin D:Cdk4/6-dependent mono-phosphorylation of Rb being inhibitory to E2F. Under this condition, we observed that the length of G1 phase is controlled by the rate of synthesis of Cyclin E and Emi1. This provides an explanation for the observation that overexpression of Cyclin D does not significantly alter the length of G1[31]. However, increasing the rate of Myc-dependent synthesis of Cyclin E and E2F make Cyclin D dispensable for the G0 to G1 transition as observed in some cellular contexts [51, 52]. The entry is delayed and the activity of E2F (free E2F) shows a delay relative to the total concentration of E2F (**S3A Fig**). Such a delay has been observed experimentally using a reporter for the E2F activity [53].

In Cyclin D dependent situation, we also showed that the point in G1 when cells become independent of Cyclin D is determined by CKI levels. This point coincides (at low CKI levels) or differs (at high CKI levels) with the point in G1 when cells become independent of mitogen(R-point). A difference can emerge since Cyclin D is stable after mitogen withdrawal until S-phase entry. The slow response time ascertains alleviation of the CKI barrier even in the absence of stimulus. We propose that such a scenario exists in cycling cells due to the endogenous stress in the S-phase [21, 30] or in cells emerging out of quiescence after longer treatment of mitogen withdrawal, both resulting in high CKI levels. Our analysis provides insights into the experimental observations obtained with mitogen withdrawal, anti-cyclin D treatment and Cdk4/6 inhibition in cells emerging out of quiescence and in cycling cells [30, 47]. We also showed that increasing mitogen levels overcome the effect of increase in CKI and promote the cell cycle entry (**Fig 4B**). Such a picture explains the observation that longer treatment of mitogen withdrawal requires stronger re-stimulation to exit quiescence[33]. This view is also consistent with the observation that mitogen and DNA-damage mediated signalling compete in G2 to control the cell cycle of daughter cells[30].

At the G1/S boundary, we showed that a double negative feedback loop regulation between APC/C^Cdh1^ and Emi1 could make the transition bistable with respect to Cyclin E:Cdk2 (**Fig 5**). E2F-dependent accumulation of Emi1 is insufficient to explain its timely accumulation in the late G1 phase and its action only after APC/C^Cdh1^ is initially inactivated by Cyclin E:Cdk2. Further, Emi1 overexpression accelerates APC/C^Cdh1^ inactivation suggesting that its levels should be controlled for the timely inactivation of APC/C^Cdh1^[23, 24]. A delayed accumulation of Emi1 is achieved by considering Emi1 as an APC/C^Cdh1^ substrate. Although Emi1 acts as a pseudosubstrate of APC/C^Cdh1^[54], evidences indicate that it can act as a substrate or inhibitor depending on the redox status and contains KEN and D-box motifs that are required for APC/C-dependent degradation[40]. Further, it is also shown that mRNAs of Emi1 and Cyclin A accumulate in the late G1 phase[24, 40]. However, proteins fail to accumulate in the anti-oxidant treated cells. This suggests a coordination of reactive oxygen species (ROS) production and metabolism in driving the G1/S transition. Emi1 is also one of the most pronounced translationally-repressed genes and relieving its repression is also crucial for its accumulation[55]. These experiments suggest Emi1 levels are controlled both at the level of its synthesis and degradation for the G1/S transition. Our model predicts that a feedback loop regulation of Emi1 is required for a rapid and switch-like inactivation of APC/C^Cdh1^ at the G1/S boundary.

The inactivation of APC/C^Cdh1^ is accompanied by the degradation of CKI (**Fig 2A**), which prevents stress mediated exit to quiescence (**Fig 2D and S4 Fig**). This explains the experimental observation that stress can induce exit to quiescence until APC/C^Cdh1^ is inactivated in MCF10A cells[23]. CKI degradation in S-phase also prevents DNA re-replication[43]. A recent study shows that CRL4^Cdt2^ is a major ubiquitin ligase involved in CKI degradation [21] and its activation depends on the S-phase entry[41]. A direct evidence connecting the activation of CRL4^Cdt2^ to APC/C^Cdh1^ inactivation is lacking, which has to be explored experimentally.

Further, we showed that a distinct Cdk2 requirement for R-point (low) and APC/C^Cdh1^ inactivation (high) creates a window of opportunity for stress mediated exit to quiescence after R-point passage. In cycling cells, we proposed that the Cdk2 activity is at an intermediate level between two thresholds (for Rb hyper-phosphorylation and APC/C^Cdh1^ inactivation) at the mitotic exit since early G1 phase events are shifted to G2 and APC/C^Cdh1^ is inactivated immediately. Therefore, two independent routes for proliferation to quiescence depending on the mitogen withdrawal (which affects transcriptional program) and replication stress (which affect CKI levels) exist.

Previously, mathematical models were proposed to account for the dynamics of mammalian cell cycle in full or in parts[11, 21, 25, 46, 56, 57]. The cell cycle models combined Rb-E2F and APC/C^Cdh1^ through E2F dependent synthesis of Cyclin A that promotes S-phase entry by inactivating APC/C^Cdh1^[46, 56]. However, the kinetics of APC/C^Cdh1^ inactivation is unaffected after Cyclin A knockdown in MCF10A and HeLA cells[23]. A mathematical model proposed for proliferation-quiescence decision incorporates Emi1 dependent regulation of APC/C^Cdh1^, but relies on Cyclin A:Cdk2 for the switch-like inactivation of APC/C^Cdh1^[57]. This model shows that p21 degradation in S-phase reduces the ability of cells to enter quiescence in response to endogenous DNA damage, but it occurs independent of APC/C^Cdh1^ inactivation. However, our model accounts for the experimental findings in MCF10A cells that Emi1 is required for the rapid and irreversible inactivation of APC/C^Cdh1^ [23]. We demonstrated that the inactivation of APC/C^Cdh1^ promotes CKI degradation to suppress the stress sensitivity. A variation in the regulation can be observed due to the cell-type differences. Further, we also studied the effect of Cyclin D on R-point in different cellular contexts incorporating recent experimental findings[13, 30]. In summary, our study provides mechanistic insights into both mitogen and stress sensitivity of the mammalian cell cycle. It will be interesting to test the model hypotheses by experiments to further understand the reversible transition between quiescence and proliferation.

## Supporting information

S1 Fig The mechanism of Rb phosphorylation and dephosphorylation. (TIF)

S2 Fig The mechanism of APC/C^Cdh1^ activation and inactivation. (TIF)

S3 Fig Temporal dynamics of quiescence to proliferation in the presence of mitogen and Myc- dependent synthesis of E2F (k_se2fm_ = 0.0015) and Cyclin E (k_scycem_ = 0.0005). **(a)** Wild type and **(b)** in the absence of Cyclin D (k_scycdm_ = 0, k_scycds_ = 0). Simulations are shown for S = 1 (mitogen level).
(TIF)

S4 Fig Temporal dynamics of quiescence to proliferation in the presence of stress after APC/C^Cdh1^ inactivation. The arrow represents the time of exposure to stress (k_scki_ = 0.6). Simulations are shown for S = 1 (mitogen level).
(TIF)

S5 Fig The sensitivity of Rb inactivation/E2F activation threshold to 5-fold change in parameter values. This plot is obtained by performing two parameter bifurcation analyses of Rb-E2F bistable switch (Fig 4). The shift in the saddle node corresponding to Rb inactivation/E2F activation threshold is shown for 5 fold increase/decrease in base parameter value (**S1 Supporting information**). The dashed lines (—) indicate the two fold change in the threshold and dotted lines (…) indicate the normalized base parameter value and threshold.
(TIF)

S6 Fig The sensitivity of APC/C^Cdh1^ inactivation threshold to 5-fold change in parameter values. This plot is obtained by performing two parameter bifurcation analyses of APC/C^Cdh1^-Emi1 bistable switch (Fig 5). The shift in the saddle node corresponding to APC/C^Cdh1^ inactivation threshold is shown for 5 fold increase/decrease in base parameter value (**S1 Supporting information**). The dashed lines (--) indicate the two fold change in the threshold and dotted lines (…) indicate the normalized base parameter value and threshold.
(TIF)

S1 Supporting information Model description and XPPAUT codes. (PDF)
