# Supplementary figures and images for: Mathematical modelling of reversible transition between quiescence and proliferation
Source: PLoS One. 2018 Jun 1;13(6):e0198420. doi: 10.1371/journal.pone.0198420 (PMC5983510; doi:10.1371/journal.pone.0198420)

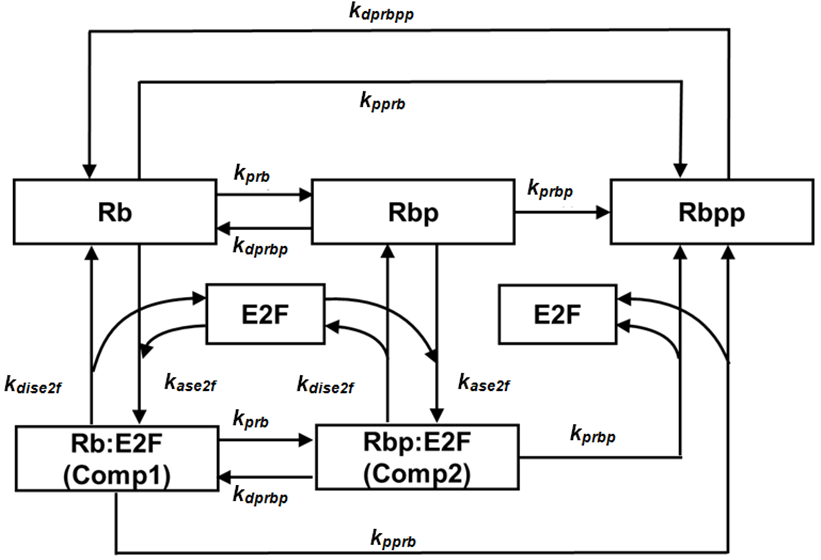

Supplement: S1 Fig — (TIF) [file pone.0198420.s001.tif]

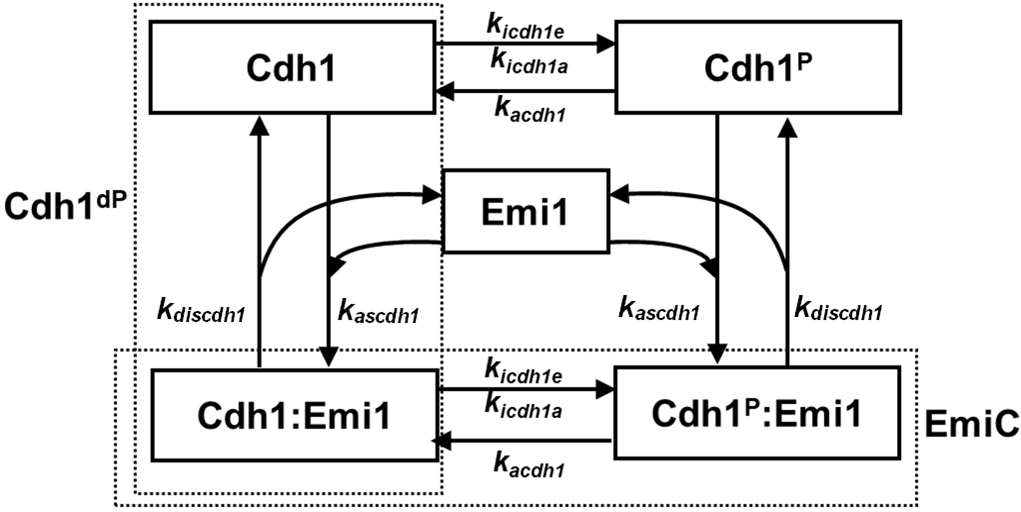

Supplement: S2 Fig — (TIF) [file pone.0198420.s002.tif]

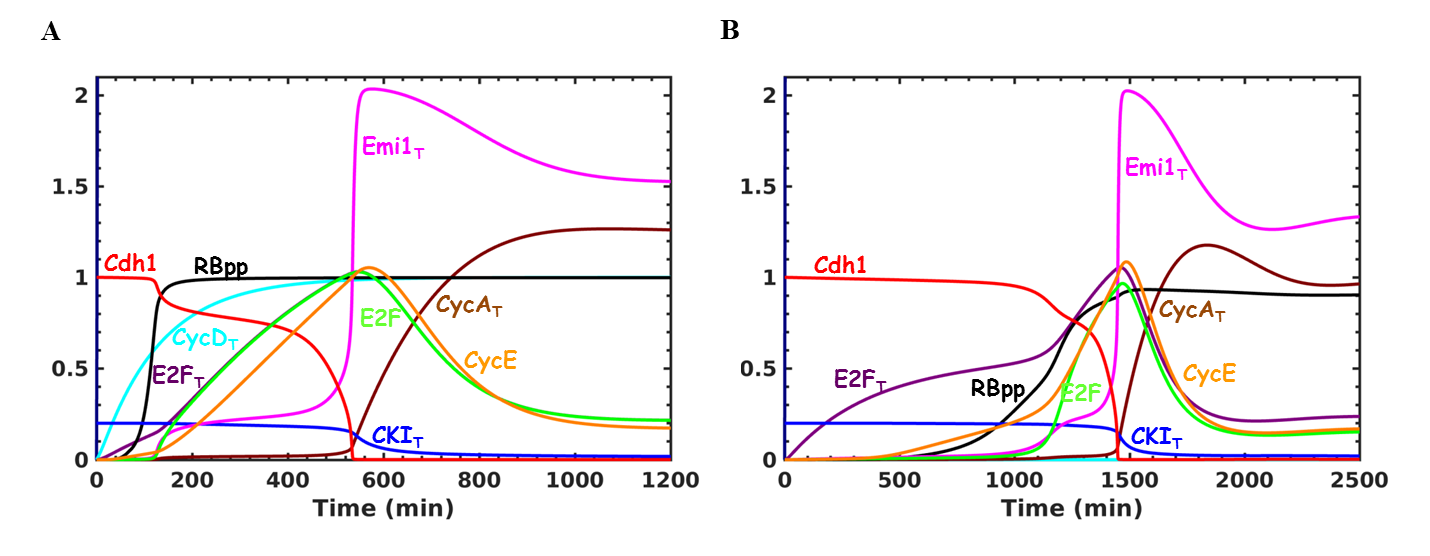

Supplement: S3 Fig — (a) Wild type and (b) in the absence of Cyclin D (kscycdm = 0, kscycds = 0). Simulations are shown for S = 1 (mitogen level). (TIF) [file pone.0198420.s003.tif]

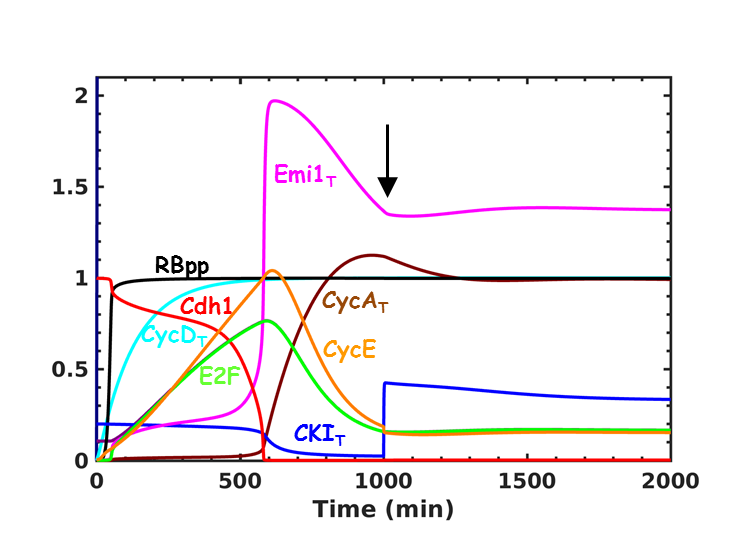

Supplement: S4 Fig — The arrow represents the time of exposure to stress (kscki = 0.6). Simulations are shown for S = 1 (mitogen level). (TIF) [file pone.0198420.s004.tif]

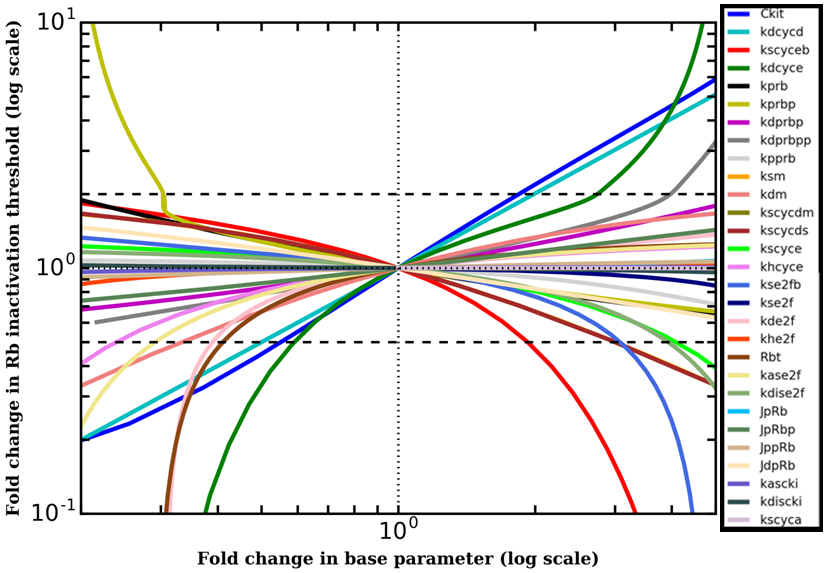

Supplement: S5 Fig — This plot is obtained by performing two parameter bifurcation analyses of Rb-E2F bistable switch (Fig 4). The shift in the saddle node corresponding to Rb inactivation/E2F activation threshold is shown for 5 fold increase/decrease in base parameter value (S1 Supporting information). The dashed lines (—) indicate the two fold change in the threshold and dotted lines (…) indicate the normalized base parameter value and threshold. (TIF) [file pone.0198420.s005.tif]

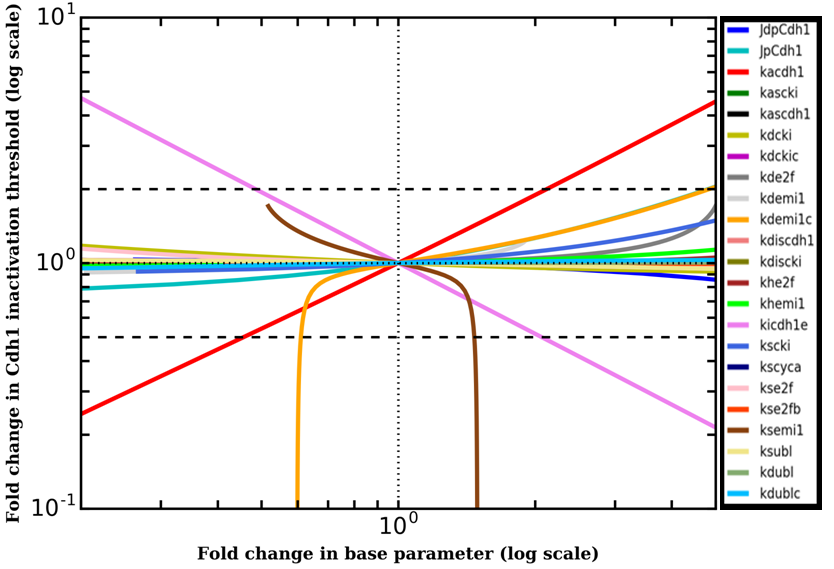

Supplement: S6 Fig — This plot is obtained by performing two parameter bifurcation analyses of APC/CCdh1-Emi1 bistable switch (Fig 5). The shift in the saddle node corresponding to APC/CCdh1 inactivation threshold is shown for 5 fold increase/decrease in base parameter value (S1 Supporting information). The dashed lines (--) indicate the two fold change in the threshold and dotted lines (…) indicate the normalized base parameter value and threshold. (TIF) [file pone.0198420.s006.tif]
