# Supplementary material for: Mathematical modelling of reversible transition between quiescence and proliferation
Source: PLoS One. 2018 Jun 1;13(6):e0198420. doi: 10.1371/journal.pone.0198420 (PMC5983510; doi:10.1371/journal.pone.0198420)
Supplement: S1 Supporting information — (PDF) [file pone.0198420.s007.pdf]

Supporting information

Mathematical modelling of reversible transition between quiescence and proliferation

Nishtha Pandey and P.K. Vinod*

Center for Computational Natural Sciences and Bioinformatics, International Institute of Information Technology, Hyderabad-500032, India

Model of the reversible transition between quiescence and proliferation

The key components of the regulatory network that are involved in the reversible transition between quiescence and proliferation include cyclin dependent kinases Cyclin D: Cdk4/6, Cyclin E:Cdk2 and Cyclin A:Cdk2, transcriptional factors Myc and E2F, Cdk inhibitor CKI, E2F inhibitor Rb and ubiquitin ligase APC/C^{Cdh1}. The dynamics of individual components is described by ordinary differential equation (ODE). All dynamic variables are dimensionless and represent relative protein concentrations. Unless stated otherwise, the synthesis, degradation, association and dissociation reactions are represented by law of mass action. E2F dependent synthesis of E2F, Cyclin E, Cyclin A and Emi1 are described by Hill equation. We used Hill coefficient equal to 1 assuming no cooperativity in gene activation. This kinetics helps to attain the state of saturation, a frequently observed scenario in biological systems. Moreover, the half saturation constant measures the binding affinity of transcription factor to promoter and is useful in distinguishing the dynamics of proteins which are regulated by a common transcriptional factor. The multi-site phosphorylation and dephosphorylation of APC/C^{Cdh1} and Rb is described by Michaelis-Menten kinetics. Such kinetics has been used previously to model Cdh1 and Rb[1,2]. The multi-site phosphorylation of proteins is shown to make the response ultrasensitive[3] and is described by Goldbeter-Koshland kinetics, where the forward and backward reactions have Michaelis-Menten kinetics[4].

Rate constants (k) have a dimension of min^{-1}. Michaelis constants (J), Half-saturation constants and other parameters are dimensionless. The subscripts of k represent the type of reaction (s, synthesis; d, degradation; a, activation; i, inactivation; as, association; dis, dissociation; h, half-saturation constants, p, phosphorylation; dp, dephosphorylation) and an abbreviation of the protein name. The parameter values were initially guessed and then refined by comparing the numerical simulations to single cell cycle dynamics of Rb hyper-phosphorylation, E2F and APC/C activity [5-7]. We also captured the behaviour under perturbations: siRNA (Cyclin E, Emi1 and Cyclin A), anti-Cyclin D (antibody), Emi1 overexpression and Cdk inhibitors[5,8-10]. Further, we performed one and two parameter bifurcation analyses to characterize the effect of different parameters on the threshold for Rb hyper-phosphorylation and APC/C^{Cdh1} inactivation (**S5 and S6 Figs**). The complete model including equations and parameters are listed in **S1-S4 Tables**. We also provide the XPPAUT code that can be used to simulate all the figures in the manuscript. XPPAUT is freely available software from G. Bard Ermentrout (Department of Mathematics, University of Pittsburgh, PA, USA) website: http://www.math.pitt.edu/~bard/xpp/xpponw95.html).

Figure 4 is generated from the full code using mitogen (S) and CKI total as bifurcation parameters and in the absence of Cyclin A (to eliminate the effect of negative feedback loop: E2F and Cyclin E degradation by Cyclin A:Cdk2 and double negative feedback loop: Rb hyper-phosphorylation by Cyclin A:Cdk2). **Figure 4b** is generated using CKI total as a second bifurcation parameter. **Figure 5** is generated from the full code using S=1 with Cyclin E total as a bifurcation parameter in the absence of Rb-E2F switch (Rbt=0) and Cyclin A to study the effect of the feedback loop between APC/C^{Cdh1} and Emi1. In the parameter sensitivity analysis, we show the effect of synthesis rate of Cyclin A on the threshold for Rb hyper-phosphorylation and APC/C^{Cdh1} inactivation (**S5 and S6 Figs**).

S1 Table: Differential Equations

$\frac{d[\text{Myc}]}{dt} = k_{sm} * [\text{S}] - k_{dm} * [\text{Myc}]$	(1)
$\frac{d[\text{CycD}_\text{T}]}{dt} = k_{scycdm} * [\text{Myc}] + k_{scycds} * [\text{S}] - k_{dcycd} * [\text{CycD}_\text{T}]$	(2)
$\frac{d[\text{CycE}_\text{T}]}{dt} = k_{scyceb} + k_{scycem} * [\text{Myc}] + k_{scyce} * \frac{[\text{E2F}]}{(k_{hcyce} + [\text{E2F}])} - (k_{dcyce} + k_{dcycea} * [\text{CycA}]) * [\text{CycE}_\text{T}]$	(3)
$\frac{d[\text{CycA}_\text{T}]}{dt} = k_{scyca} * \frac{[\text{E2F}]}{(k_{hcyca} + [\text{E2F}])} - (k_{dcyca} + k_{dcycac} * [\text{Cdh1}]) * [\text{CycA}_\text{T}]$	(4)
$\frac{d[\text{E2F}_\text{T}]}{dt} = k_{se2fb} + k_{se2fm} * [\text{Myc}] + k_{se2f} * \frac{[\text{E2F}]}{(k_{he2f} + [\text{E2F}])} - (k_{de2f} + k_{de2fa} * [\text{CycA}]) * [\text{E2F}_\text{T}]$	(5)
$\frac{d[\text{Comp1}]}{dt} = k_{ase2f} * [\text{E2F}] * [\text{Rb}] + k_{dprbp} * \frac{[\text{Comp2}]}{(J_{dpRb} + [\text{Comp2}])} - k_{dise2f} * [\text{Comp1}] - k_{prb} * [\text{CycD}] * \frac{[\text{Comp1}]}{(J_{pRb} + [\text{Comp1}])} - k_{pprb} * ([\text{CycE}] + [\text{CycA}]) * \frac{[\text{Comp1}]}{(J_{ppRb} + [\text{Comp1}])} - (k_{de2f} + k_{de2fa} * [\text{CycA}]) * [\text{Comp1}]$	(6)
$\frac{d[\text{Comp2}]}{dt} = k_{ase2f} * [\text{E2F}] * [\text{Rbp}] + k_{prb} * [\text{CycD}] * \frac{[\text{Comp1}]}{(J_{pRb} + [\text{Comp1}])} - k_{dise2f} * [\text{Comp2}] - k_{dprbp} * \frac{[\text{Comp2}]}{(J_{dpRb} + [\text{Comp2}])} - k_{prbp} * ([\text{CycE}] + [\text{CycA}]) * \frac{[\text{Comp2}]}{(J_{pRbp} + [\text{Comp2}])} - (k_{de2f} + k_{de2fa} * [\text{CycA}]) * [\text{Comp2}]$	(7)
$\frac{d[\text{Rbp}]}{dt} = k_{prb} * [\text{CycD}] * \frac{[\text{Rb}]}{(J_{pRb} + [\text{Rb}])} + k_{dise2f} * [\text{Comp2}] + (k_{de2f} + k_{de2fa} * [\text{CycA}]) * [\text{Comp2}] - k_{ase2f} * [\text{E2F}] * [\text{Rbp}] - k_{dprbp} * \frac{[\text{Rbp}]}{(J_{dpRb} + [\text{Rbp}])} - k_{prbp} * ([\text{CycE}] + [\text{CycA}]) * \frac{[\text{Rbp}]}{(J_{pRbp} + [\text{Rbp}])}$	(8)

$$\frac{d[\mathrm{Rbpp}]}{dt} = k_{prbp} * ([\mathrm{CycE}] + [\mathrm{CycA}]) * \frac{[\mathrm{Rbp}]}{\left(J_{pRbp} + [\mathrm{Rbp}]\right)} + k_{prbp} * ([\mathrm{CycE}] + [\mathrm{CycA}]) * \frac{[\mathrm{Comp2}]}{\left(J_{pRbp} + [\mathrm{Comp2}]\right)} + k_{pprb} * ([\mathrm{CycE}] + [\mathrm{CycA}]) * \frac{[\mathrm{Rb}]}{\left(J_{ppRb} + [\mathrm{Rb}]\right)} + k_{pprb} * ([\mathrm{CycE}] + [\mathrm{CycA}]) * \frac{[\mathrm{Comp1}]}{\left(J_{ppRb} + [\mathrm{Comp1}]\right)} - k_{dprbpp} * \frac{[\mathrm{Rbpp}]}{\left(J_{dpRb} + [\mathrm{Rbpp}]\right)}$$	(9)
$$\frac{d[\mathrm{Cki_T}]}{dt} = k_{scki} - (k_{dcki} + k_{dckic} * ([\mathrm{CycE}] + [\mathrm{CycA}]) * [\mathrm{Ubl}]) * [\mathrm{Cki_T}]$$	(10)
$$\frac{d[\mathrm{CycECki}]}{dt} = k_{ascki} * [\mathrm{CycE}] * ([\mathrm{Cki_T}] - [\mathrm{CycECki}] - [\mathrm{CycDCki}] - [\mathrm{CycACki}]) - \left(k_{discki} + k_{dcyce} + k_{dcycea} * [\mathrm{CycA}] + k_{dcki} + k_{dckic} * ([\mathrm{CycE}] + [\mathrm{CycA}]) * [\mathrm{Ubl}]\right) * [\mathrm{CycECki}]$$	(11)
$$\frac{d[\mathrm{CycDCki}]}{dt} = k_{ascki} * [\mathrm{CycD}] * ([\mathrm{Cki_T}] - [\mathrm{CycECki}] - [\mathrm{CycDCki}] - [\mathrm{CycACki}]) - \left(k_{discki} + k_{dcycd} + k_{dcki} + k_{dckic} * ([\mathrm{CycE}] + [\mathrm{CycA}]) * [\mathrm{Ubl}]\right) * [\mathrm{CycDCki}]$$	(12)
$$\frac{d[\mathrm{CycACki}]}{dt} = k_{ascki} * [CycA] * ([\mathrm{Cki_T}] - [\mathrm{CycECki}] - [\mathrm{CycDCki}] - [\mathrm{CycACki}]) - \left(k_{discki} + k_{dcyca} + k_{dcycac} * [\mathrm{Cdh1}] + k_{dcki} + k_{dckic} * ([\mathrm{CycE}] + [\mathrm{CycA}]) * [Ubl]\right) * [\mathrm{CycACki}]$$	(13)
$$\frac{d[\mathrm{Cdh1dp}]}{dt} = k_{acdh1} * \frac{([\mathrm{Cdh1_T}] - [\mathrm{Cdh1dp}])}{\left(J_{dpCdh1} + ([\mathrm{Cdh1_T}] - [\mathrm{Cdh1dp}])\right)} - (k_{icdh1e} * [\mathrm{CycE}] * + k_{icdh1a} * [\mathrm{CycA}]) * \frac{[\mathrm{Cdh1dp}]}{\left(J_{pCdh1} + [\mathrm{Cdh1dp}]\right)}$$	(14)
$$\frac{d[\mathrm{Emi1_T}]}{dt} = k_{semi1} * \frac{[\mathrm{E2F}]}{(k_{hemi1} + [\mathrm{E2F}])} - (k_{demi1} + k_{demi1c} * [\mathrm{Cdh1}]) * [\mathrm{Emi1_T}]$$	(15)
$$\frac{d[\mathrm{EmiC}]}{dt} = k_{ascdh1} * ([\mathrm{Cdh1_T}] - [\mathrm{EmiC}]) * ([\mathrm{Emi1_T}] - [\mathrm{EmiC}]) - (k_{discdh1} + k_{demi1} + k_{demi1c} * [\mathrm{Cdh1}]) * [\mathrm{EmiC}]$$	(16)
$$\frac{d[\mathrm{Cdh1}]}{dt} = k_{acdh1} * \frac{([\mathrm{Cdh1_T}] - [\mathrm{EmiC}] - [\mathrm{Cdh1}])}{\left(J_{dpCdh1} + ([\mathrm{Cdh1_T}] - [\mathrm{Cdh1dp}])\right)} + (k_{discdh1} + k_{demi1} + k_{demi1c} * [\mathrm{Cdh1}]) * ([\mathrm{Cdh1dp}] - [\mathrm{Cdh1}]) - (k_{icdh1e} * [\mathrm{CycE}] + k_{icdh1a} * [\mathrm{CycA}]) * \frac{[\mathrm{Cdh1}]}{\left(J_{pCdh1} + [\mathrm{Cdh1dp}]\right)} - k_{ascdh1} * [\mathrm{Cdh1}] * ([\mathrm{Emi1_T}] - [\mathrm{EmiC}])$$	(17)

$$\frac{d[\mathrm{Ubl}]}{dt} = k_{subl} - (k_{dubl} + k_{dublc} * [\mathrm{Cdh1}]) * [\mathrm{Ubl}]$$	(18)

S2 Table: Algebraic Equations

$[\mathrm{E2F}] = [\mathrm{E2F_T}] - [\mathrm{Comp1}] - [\mathrm{Comp2}]$	(19)
$[\mathrm{Rb}] = [\mathrm{Rb_T}] - [\mathrm{Rbp}] - [\mathrm{Rbpp}] - [\mathrm{Comp1}] - [\mathrm{Comp2}]$	(20)
$[\mathrm{CycE}] = [\mathrm{CycE_T}] - [\mathrm{CycECki}]$	(21)
$[\mathrm{CycD}] = [\mathrm{CycD_T}] - [\mathrm{CycDCki}]$	(22)
$[\mathrm{CycA}] = [\mathrm{CycA_T}] - [\mathrm{CycACki}]$	(23)
$[\mathrm{Cdh1^P}] = [\mathrm{Cdh1_T}] - [\mathrm{Cdh1}] - [\mathrm{EmiC}]$	(24)

S3 Table: Model parameter description

Symbol	Description	Value
S	Mitogen level	1
k_{sm}	Rate constant of Myc synthesis driven by Mitogen	0.1
k_{dm}	Rate constant of Myc degradation	0.1
k_{scycdm}	Rate constant of Cyclin D synthesis driven by Myc	0.004
k_{scycds}	Rate constant of Cyclin D synthesis driven by Mitogen	0.004
k_{dcycd}	Rate constant of Cyclin D degradation	0.008
k_{scyceb}	Rate constant of basal(constitutive) Cyclin E synthesis	0.0001
k_{scycem}	Rate constant of Cyclin E synthesis driven by Myc	0
k_{scyce}	Rate constant of Cyclin E synthesis driven by E2F	0.004
k_{dcyce}	Rate constant of Cyclin E degradation	0.001
k_{dcycea}	Rate constant of Cyclin E degradation driven by Cyclin A	0.01
k_{hcyce}	Half saturation constant of E2F for Cyclin E synthesis	0.25
k_{scyca}	Rate constant of Cyclin A synthesis driven by E2F	0.008
k_{dcyca}	Rate constant of Cyclin A degradation	0.004
k_{dcycac}	Rate constant of Cyclin A degradation driven by APC/C^{Cdh1}	0.5
k_{hcyca}	Half saturation constant of E2F for Cyclin A synthesis	0.1
k_{se2fb}	Rate constant of basal(constitutive) E2F synthesis	0.0003
k_{se2fm}	Rate constant of E2F synthesis driven by Myc	0
k_{se2f}	Rate constant of auto-regulated E2F synthesis	0.004
k_{de2f}	Rate constant of E2F degradation	0.003
k_{de2fa}	Rate constant of E2F degradation driven by Cyclin A	0.01
k_{he2f}	Half saturation constant of E2F for its synthesis	0.25
Rb_T	Total concentration of Rb	1
k_{ase2f}	Association rate constant of E2F and Rb; E2F and Rbp	100
k_{dise2f}	Dissociation rate constant of Comp1 and Comp2	1
k_{prb}	Phosphorylation rate constant of Rb/Comp1 by Cyclin D:Cdk4/6	5
k_{dprbp}	Dephosphorylation rate constant of Rbp/Comp2	1
k_{pprb}	Phosphorylation rate constant of Rb/Comp1 by Cyclin E:Cdk2	0.5
k_{dprbpp}	Dephosphorylation rate constant of Rbpp	0.1
k_{prbp}	Phosphorylation rate constant of Rbp/Comp2 by Cyclin E:Cdk2	2
J_{pRb}	Michaelis constant for phosphorylation of Rb/Comp1	0.01
J_{pRbp}	Michaelis constant for hyper-phosphorylation of Rbp/Comp2	0.05
J_{dpRb}	Michaelis constant for dephosphorylation of Rbp/Comp2/Rbpp	0.05
J_{ppRb}	Michaelis constant for hyper-phosphorylation of Rb/Comp1	0.5
k_{scki}	Rate constant of basal(constitutive) CKI synthesis	0.04
k_{dcki}	Rate constant of CKI degradation	0.2

k_{dckic}	Rate constant of CKI degradation driven by ubiquitin ligase	1
k_{ascki}	Association rate constant of CKI and Cdk	100
k_{discki}	Dissociation rate constant of CKI:Cdk complex	0.1
k_{subl}	Rate constant of basal(constitutive) ubiquitin ligase synthesis	0.004
k_{dubl}	Rate constant of ubiquitin ligase degradation	0.002
k_{dublc}	Rate constant of ubiquitin ligase degradation driven by APC/C^{Cdh1}	0.2
k_{semi1}	Rate constant of Emi1 synthesis driven by E2F	0.45
k_{demi1}	Rate constant of Emi1 degradation	0.2
k_{demi1c}	Rate constant of Emi1 degradation driven by APC/C^{Cdh1}	2
k_{hemi1}	Half saturation constant of E2F for Emi1 synthesis	0.1
k_{ascdh1}	Association rate constant of Emi1 and APC/C^{Cdh1}	100
$k_{discdh1}$	Dissociation rate constant of Emi1:APC/C^{Cdh1} complex	0.1
$Cdh1_T$	Total concentration of APC/C^{Cdh1}	1
k_{acdh1}	Rate constant for activation of APC/C^{Cdh1} by dephosphorylation	0.1
k_{icdh1e}	Rate constant for inactivation of APC/C^{Cdh1} by Cyclin E:Cdk2	0.14
k_{icdh1a}	Rate constant for inactivation of APC/C^{Cdh1} by Cyclin A:Cdk2	0.2
J_{dpCdh1}	Michaelis constant for APC/C^{Cdh1} dephosphorylation	0.02
J_{pCdh1}	Michaelis constant for APC/C^{Cdh1} phosphorylation by CycE:Cdk2 and CycA:Cdk2	0.25

S4 Table: Dynamic variable description

Symbol	Description	Initial Condition
[Myc]	Concentration of Myc	0
$[CycD_T]$	The total concentration of Cyclin D	0
$[CycE_T]$	The total concentration of Cyclin E	0.119
$[CycA_T]$	The total concentration of Cyclin A	0
$[E2F_T]$	The total concentration of E2F transcription factor	0.106
[Comp1]	Concentration of Rb:E2F complex [Fig. S1]	0.105
[Comp2]	Concentration of Rbp:E2F complex [Fig. S1]	0
[Rbp]	Mono-phosphorylated Rb [Fig. S1]	0
[Rbpp]	Hyper-phosphorylated Rb [Fig. S1]	0.001
$[Cki_T]$	The total concentration of cyclin dependent kinase (CDK) inhibitors	0.2
[CycECki]	Concentration of Cyclin E:CKI complex	0.115
[CycDCki]	Concentration of Cyclin D:CKI complex	0
[CycACki]	Concentration of Cyclin A:CKI complex	0
[Cdh1dp]	The total concentration of APC/C^{Cdh1} in dephosphorylated form (includes both free APC/C^{Cdh1} and APC/C^{Cdh1} bound to Emi1) [Fig. S2]	1
$[Emi1_T]$	The total concentration of Emi1	0.002
[EmiC]	Concentration of Emi1: APC/C^{Cdh1} complex (includes both phosphorylated and dephosphorylated APC/C^{Cdh1} in complex with Emi1) [Fig. S2]	0.002
[Cdh1]	Concentration of free, dephosphorylated APC/C^{Cdh1} [Fig. S2]	0.998
[Ubl]	The total concentration of Ubiquitin ligases	0.02
[E2F]	Concentration of free E2F	
[Rb]	Concentration of free, dephosphorylated Rb [Fig. S1]	
[CycE]	Concentration of free Cyclin E	
[CycD]	Concentration of free Cyclin D	
[CycA]	Concentration of free Cyclin A	
$[Cdh1^P]$	Concentration of free, phosphorylated APC/C^{Cdh1} [Fig. S2]	

XPPAUT code for temporal dynamics of quiescence to proliferation transition: R-point passage and G1/S transition

```
# XPPAUT code that can be copied to a text file with *.ode extension
# Cyclin D dependent cell cycle entry: Default parameter values
# Cyclin D independent cell cycle entry:
kse2fm=0.0015,kscycem=0.0005,kse2fb=0,kscyceb=0. Initial conditions:
Ckit=0.2,Ubl=0.0198. All other dynamic variables start with 0
# Parameter changes done to simulate different experimental conditions :
# Mitogen starvation (S=0), Stress (kscki=0.6), anti-cyclin D (kdcycd=0.1), Cdk inhibition
(kdcyce=0.01, kdcyca=0.01)
# Emi1 deletion (ksemi1=0)

# Initial conditions for dynamic variables
init Myc=0,Cycdt=0,CycEt=0.119,CycAt=0,E2ft=0.106,comp1=0.105,comp2=0
init Rbp=0,Rbpp=0.001,Ckit=0.2,CycECki=0.115,CycDCki=0, CycACki=0,Cdh1dp=1
init Emi1T=0.002,EmiC=0.002,Cdh1=0.998,Ubl=0.02

# Values of kinetic parameter (parameter description in Table S3)
par S=1,ksm=0.1,kdm=0.1,kscycdm=0.004,kscycds=0.004,kdcycd=0.008
par kscyceb=0.0001,kscycem=0,kscyce=0.004,kdcyce=0.001,kdcycea=0.01
par khcyce=0.25,kscyca=0.008,kdcyca=0.004,kdcycac=0.5,khcyca=0.1
par kse2fb=0.0003,kse2fm=0,kse2f=0.004,kde2f=0.003,kde2fa=0.01
par khe2f=0.25,RBt=1
par kase2f=100,kdise2f=1
par kprb=5,kprbp=2,kpprb=0.5,kdprbp=1,kdprbpp=0.1
par JpRb=0.01,JpRbp=0.05,JppRb=0.5,JdpRb=0.05
par kscki=0.04,kdcki=0.2,kdckic=1
par kascki=100,kdiscki=0.1
par ksubl=0.004,kdubl=0.002,kdublc=0.2
par ksemi1=0.45,kdemi1=0.2,kdemi1c=2,khemi1=0.1
par kascdh1=100,kdiscdh1=0.1
par Cdh1T=1,kacdh1=0.1,kicdh1e=0.14,kicdh1a=0.2,JdpCdh1=0.02,JpCdh1=0.25

# Set of differential equations:
# Mitogen (S) dependent synthesis of Myc
Myc' = ksm*S - kdm*Myc

# Cycdt represents the total concentration of Cyclin D
Cycdt' = kscycdm*Myc + kscycds*S - kdcycd*Cycdt

# CycEt represents the total concentration of Cyclin E
CycEt' = kscyceb + kscycem*Myc + kscyce*E2f/(khcyce + E2f) - (kdcyce +
kdcycea*CycA)*CycEt

# CycAt represents the total concentration of Cyclin A
CycAt' = kscyca*E2f/(khcyca + E2f) - (kdcyca + kdcycac*Cdh1)*CycAt

# E2ft represents the total concentration of E2F
E2ft' = kse2fb + kse2fm*Myc + kse2f*E2f/(khe2f + E2f) - (kde2f + kde2fa*CycA)*E2ft
```

```
# comp1 represents complex between free E2F and Rb
comp1' = kase2f*E2f*Rb + kdprbp*comp2/(JdpRb + comp2) - kdise2f*comp1 -
kprb*Cycd*comp1/(JpRb + comp1) - kpprb*(CycE + CycA)*comp1/(JppRb + comp1) -
(kde2f + kde2fa*CycA)*comp1

# comp2 represents complex between free E2F and Rb in mono-phosphorylated form
comp2' = kase2f*E2f*Rbp + kprb*Cycd*comp1/(JpRb + comp1) - kdise2f*comp2 -
kdprbp*comp2/(JdpRb+comp2) - kprbp*(CycE + CycA)*comp2/(JpRbp + comp2) - (kde2f +
kde2fa*CycA)*comp2

# Rbp represents mono-phosphorylated Rb (active form)
Rbp' = kprb*Cycd*Rb/(JpRb + Rb) + kdise2f*comp2  - kase2f*E2f*Rbp -
kdprbp*Rbp/(JdpRb + Rbp) - kprbp*(CycE + CycA)*Rbp/(JpRbp + Rbp) + (kde2f +
kde2fa*CycA)*comp2

# Rbpp represents hyper-phosphorylated Rb (inactive form)
Rbpp' = kprbp*(CycE + CycA)*Rbp/(JpRbp + Rbp) + kprbp*(CycE + CycA)*comp2/(JpRbp
+ comp2) + kpprb*(CycE + CycA)*Rb/(JppRb + Rb) + kpprb*(CycE +
CycA)*comp1/(JppRb + comp1) - kdprbpp*Rbpp/(JdpRb + Rbpp)

# Ckit represents the total concentration of CKI
Ckit' = kscki - (kdcki + kdckic*(CycE + CycA)*Ubl)*Ckit

# CycECki represents CKI in complex with Cyclin E
CycECki' = kascki*CycE*(Ckit-CycECki-CycDCki-CycACki) - (kdiscki + kdcyce +
kdcycea*CycA + kdcki + kdckic*(CycE + CycA)*Ubl)*CycECki

# CycDCki represents CKI in complex with Cyclin D
CycDCki' = kascki*CycD*(Ckit-CycECki-CycDCki-CycACki) - (kdiscki + kdcycd + kdcki
+ kdckic*(CycE + CycA)*Ubl)*CycDCki

# CycACki represents CKI in complex with Cyclin A
CycACki' = kascki*CycA*(Ckit-CycECki-CycDCki-CycACki) - (kdiscki + (kdcyca +
kdcycac*Cdh1) + kdcki + kdckic*(CycE + CycA)*Ubl)*CycACki

# Cdh1dp represents total dephosphorylated forms: free and in complex with Emi1 (fig. S2)
Cdh1dp' = kacdh1*(Cdh1T-Cdh1dp)/(JdpCdh1 + (Cdh1T-Cdh1dp)) - (kicdh1e*CycE+
kicdh1a*CycA )*Cdh1dp/(JpCdh1 + Cdh1dp)

# Emi1T represents the total concentration of Emi1
Emi1T' = ksemi1*E2f/(khemi1 + E2f) - (kdemi1 + kdemi1c*Cdh1)*Emi1T

# Emi1C represents Emi1 in complex with both phosphorylated and dephosphorylated Cdh1
(fig. S2)
EmiC' = kascdh1*(Cdh1T - EmiC)*(Emi1T-EmiC) - (kdiscdh1 + kdemi1 +
kdemi1c*Cdh1)*EmiC

# Cdh1 represents free active dephosphorylated form of Cdh1 (fig. S2)
```

```
Cdh1' = kacdh1*(Cdh1T-EmiC-Cdh1)/(JdpCdh1 + (Cdh1T-Cdh1dp)) + (kdiscdh1 + kdemi1
+ kdemi1c*Cdh1)*(Cdh1dp-Cdh1) - (kicdh1e*CycE + kicdh1a*CycA)*Cdh1/(JpCdh1 +
Cdh1dp) - kascdh1*(Emi1T-EmiC)*Cdh1

# Ubl represents ubiquitin ligase
Ubl' = ksubl - (kdubl + kdublc*Cdh1)*Ubl


# Set of algebraic expression:
# E2f is free form of the E2F
E2f = E2ft - comp1 - comp2
aux E2f = E2ft - comp1 - comp2

# Rb is active, dephosphorylated form of Rb
Rb = Rbt - comp1 - comp2 - Rbp - Rbpp
aux Rb = Rbt - comp1 - comp2 - Rbp - Rbpp

# CycE is free form of Cyclin E
CycE = CycEt - CycECki
aux CycE = CycEt - CycECki

# CycD is free form of Cyclin D
Cycd = Cycdt - CycDCki
aux Cycd = Cycdt - CycDCki

# CycA is free form of Cyclin A
CycA = CycAt - CycACki
aux CycA = CycAt - CycACki

# Cdh1p is free form of phosphorylated Cdh1
Cdh1p = Cdh1t - EmiC - Cdh1
aux Cdh1p = Cdh1t - EmiC - Cdh1

@ total=1200,dt=0.5,method=stiff
@ xlo=0,xhi=1200,ylo=0,yhi=2
@
NPLOT=8,yp1=Rbpp,yp2=Cycdt,yp3=E2ft,yp4=E2f,yp5=CycE,yp6=Ckit,yp7=Cdh1,yp8=E
mi1T
#Black=Rbpp,Red=Cycdt,Redorange=E2ft,Orange=E2f,Yelloworange=CycE,Yellow=Ckit,
Yellowgreen=Cdh1,Green=Emi1T
@ NTST=15,NMAX=20000000,NPR=1000,DS=0.02
@ DSMAX=0.05,DSMIN=0,PARMIN=0,PARMAX=3
@ AUTOXMIN=0,AUTOXMAX=3,AUTOYMIN=0,AUTOYMAX=1

done
```

References

1. Yao G, Lee TJ, Mori S, Nevins JR, You L (2008) A bistable Rb-E2F switch underlies the restriction point. Nat Cell Biol 10: 476-482.
2. Novak B, Tyson JJ (2004) A model for restriction point control of the mammalian cell cycle. J Theor Biol 230: 563-579.
3. Kapuy O, Barik D, Sananes MR, Tyson JJ, Novak B (2009) Bistability by multiple phosphorylation of regulatory proteins. Prog Biophys Mol Biol 100: 47-56.
4. Goldbeter A, Koshland DE, Jr. (1981) An amplified sensitivity arising from covalent modification in biological systems. Proc Natl Acad Sci U S A 78: 6840-6844.
5. Cappell SD, Chung M, Jaimovich A, Spencer SL, Meyer T (2016) Irreversible APC(Cdh1) Inactivation Underlies the Point of No Return for Cell-Cycle Entry. Cell 166: 167-180.
6. Spencer SL, Cappell SD, Tsai FC, Overton KW, Wang CL, et al. (2013) The proliferation-quiescence decision is controlled by a bifurcation in CDK2 activity at mitotic exit. Cell 155: 369-383.
7. Overton KW, Spencer SL, Noderer WL, Meyer T, Wang CL (2014) Basal p21 controls population heterogeneity in cycling and quiescent cell cycle states. Proc Natl Acad Sci U S A 111: E4386-4393.
8. Hitomi M, Stacey DW (1999) Cellular ras and cyclin D1 are required during different cell cycle periods in cycling NIH 3T3 cells. Mol Cell Biol 19: 4623-4632.
9. Merrick KA, Wohlbold L, Zhang C, Allen JJ, Horiuchi D, et al. (2011) Switching Cdk2 on or off with small molecules to reveal requirements in human cell proliferation. Mol Cell 42: 624-636.
10. Yang HW, Chung M, Kudo T, Meyer T (2017) Competing memories of mitogen and p53 signalling control cell-cycle entry. Nature 549: 404-408.
